# Ceramic Microbial Fuel Cells Stack: power generation in standard and supercapacitive mode

**DOI:** 10.1038/s41598-018-21404-y

**Published:** 2018-02-19

**Authors:** Carlo Santoro, Cristina Flores-Cadengo, Francesca Soavi, Mounika Kodali, Irene Merino-Jimenez, Iwona Gajda, John Greenman, Ioannis Ieropoulos, Plamen Atanassov

**Affiliations:** 1 Department of Chemical and Biological Engineering, Center for Micro-Engineered Materials (CMEM), University of New Mexico, Albuquerque, NM 87131 USA; 2 Department of Chemistry “Giacomo Ciamician”, Alma Mater Studiorum – Università di Bologna, Via Selmi, 2, 40126 Bologna, Italy; 3 Bristol BioEnergy Centre, Bristol Robotics Laboratory, T-Block, UWE, Coldharbour Lane, Bristol, BS16 1QY UK; 4 Biological, Biomedical and Analytical Sciences, UWE, Coldharbour Lane, Bristol, BS16 1QY UK

## Abstract

In this work, a microbial fuel cell (MFC) stack containing 28 ceramic MFCs was tested in both standard and supercapacitive modes. The MFCs consisted of carbon veil anodes wrapped around the ceramic separator and air-breathing cathodes based on activated carbon catalyst pressed on a stainless steel mesh. The anodes and cathodes were connected in parallel. The electrolytes utilized had different solution conductivities ranging from 2.0 mScm^−1^ to 40.1 mScm^−1^, simulating diverse wastewaters. Polarization curves of MFCs showed a general enhancement in performance with the increase of the electrolyte solution conductivity. The maximum stationary power density was 3.2 mW (3.2 Wm^−3^) at 2.0 mScm^−1^ that increased to 10.6 mW (10.6 Wm^−3^) at the highest solution conductivity (40.1 mScm^−1^). For the first time, MFCs stack with 1 L operating volume was also tested in supercapacitive mode, where full galvanostatic discharges are presented. Also in the latter case, performance once again improved with the increase in solution conductivity. Particularly, the increase in solution conductivity decreased dramatically the ohmic resistance and therefore the time for complete discharge was elongated, with a resultant increase in power. Maximum power achieved varied between 7.6 mW (7.6 Wm^−3^) at 2.0 mScm^−1^ and 27.4 mW (27.4 Wm^−3^) at 40.1 mScm^−1^.

## Introduction

Amongst all the bioelectrochemical systems, Microbial fuel cells (MFCs) have the capability of degrading organics and generate simultaneously electricity^1–4^. This aspect renders this a promising technology for substituting or rather integrating into existing energy consuming wastewater treatment^1–5^.

Several problems are currently affecting the performance of MFCs, in a way slowing down large-scale implementation^6,7^. Starting from the anode part, electron transfer mechanisms within the electroactive biofilm are not well understood and actually quite disputed within the scientific community^8–10^, and this can become even more convoluted when the system varies from a single culture to a mixed species biofilm^8–10^. The kinetics are still fairly low and need to be substantially improved^8–10^. The majority (but not all) of the anodic materials remain relatively expensive and the existing literature does not contain sufficient numbers of long term studies in which durability and robustness are considered^1,11–13^.

The next main issue found in MFCs is the slow kinetic rate at the cathode during the reduction of oxygen^14–16^. Neutral pH operating conditions negatively affect the oxygen reduction reaction that requires H^+^ and OH^−^ to transform successfully the reagents into products. Furthermore, catalysts need to be utilized to overcome reaction overpotentials and accelerate the slow kinetics^17,18^.

Current literature showed the utilization of two different kind of catalysts, one biotic and the other abiotic^17–20^. Biotic catalysts are based on enzymes (e.g. ascorbate oxidase^21–23^, laccase^24–26^ or bilirubin oxidase^27–30^) that selectively reduce oxygen to water or to single species or mixed biofilm capable to reduce a specific oxidant such as oxygen, nitrate or sulfate^31^. Unfortunately, despite enzymes are quite active, they are not durable in polluted operating conditions^32,33^. Microbial reduction catalysis is not as efficient as the reaction with abiotic catalysts, moreover, the reaction mechanisms are quite obscure and still to be understood and further improved^16^.

Abiotic catalysts are by far the most utilized in MFCs^34–36^. The most commonly used cathode catalyst is based on platinum^34^ and this choice was mainly dictated by the utilization of those electrodes in more developed and studied classical hydrogen-fed fuel cells. Platinum is a rare metal and very expensive and therefore the utilization in large scale MFC seems senseless and irrational. As time progresses, it was also demonstrated that platinum is not durable in polluted environments and then it cannot be used as a catalyst for long term high performance^37–40^. Another two alternatives have been selected and pursued over time. The first one is based on carbonaceous materials^34–36,41^ and the second one on platinum group metal-free (PGM-free) materials^34–36^. Several carbonaceous materials such as activated carbon^42–48^, carbon black^49,50^, carbon nanotubes^51,52^, graphene^53–55^, carbon nanofibers^56,57^ have been extensively investigated as cathode catalysts in MFC. Activated carbon (AC) seems to be the most viable choice due to the commercial availability at large scale, relatively low cost and positive mechanical-chemical characteristics such as durability, mechanical strength, resistance to chemical corrosion, high surface area, etc^1^. A recent review on catalyst materials for MFCs shows an increasing utilization of AC^34^. In parallel, PGM-free catalysts are capturing the attention of the scientific community due to much higher performances compared to AC despite a minor increase in capital cost. PGM-free can be also called M-N-C with M as an earth abundant transitional metal atomically dispersed such as Mn, Fe, Co, Ni and Cu and N-C as a carbonaceous rich in nitrogen carbon support. Also in this case, several examples of PGM-free catalysts are presented in literature^58–68^ with results that underline the supremacy of Fe-based catalysts compared to Co-based, Ni-based and Mn-based^69,70^. One of the main problems is the fact that large amount of catalysts are not easily commercially available and therefore the preparation of those catalysts requires time and resources.

An additional problem that MFCs and bioelectrochemical systems (BESs) encounter is the design for scaling up of the reactor in order to harvest sufficient electricity for practical applications or in order to treat large quantity of wastewater. Generally speaking, smaller the microbial fuel cell reactor is, the greater is the power output both density (express in function of the electrode geometric area) and volumetric (express in function of the reactor empty volume)^71–77^. This is due to the fact that the reactor is well designed, distance between anode and cathode are reduced therefore ohmic losses are contained, empty volumes in which fermentation might occur are avoided and anode/cathode surface area and electrode surfaces to volume ratio are well defined and optimized^71–77^. This type of small MFCs with volume smaller than 30 mL are often used in laboratory-scale experiments with other favorable operating conditions such as high working temperature^78,79^, addition of buffer to enhance solution conductivity^80,81^, utilization of easily degradable organic compounds^3,4^ and enrichment of the electroactive bacteria using specific inoculum^1^ that all contribute to enhance the performances. Consequently, in the existing literature, is easy to encounter power outputs that are between 2 and 5 Wm^−2^ in small MFCs with large anode and small cathode^71–77^ and volumetric power over 100 Wm^−3^ when the MFCs are even smaller than 30 mL^71–77^. Interestingly, volumetric power above 10000 Wm^−3^ was presented in μL empty volume MFCs^77^.

It was shown in two recent reviews that as bigger the reactor (in terms of volume) becomes, in order to accommodate more wastewater or organic waste to treat, electrodes become bigger and one or more of the above mentioned conditions are not optimized and consequently both power density and volumetric power decreased dramatically even orders of magnitude^71–73^. In the existing literature, the power outputs of reactors with empty volume greater than 1 liter are generally lower than 1 Wm^−2^ ^71–73^ and volumetric power lower than 100 Wm^−3^ ^71–73^. The research of the scaled up optimal design is still taking place with several proposed MFCs system with volume greater than 1 L proposed recently.

Another main issue related with MFC technology is the production of low power/current and low quality energy that therefore has to be harvested in a smart way^82–85^. Cell voltage is the main parameter affecting energy and power. A simple way to increase the voltage output is to connect the different MFCs in series^82–85^. This is possible only if the electrodes do not share the same electrolyte or short-circuit or shunt current might well flow between adjacent units, thus creating a local short-circuit. Instead, if the MFCs are connected in parallel, the current produced increases and in this case the electrodes can share the same electrolyte^82–85^. There are of course application scenarios where MFCs can be configured in a way such that they can be connected in series and parallel, which simultaneously increases voltage and current^86^; this allows the MFCs to be less reliant on peripheral electronics. Generally, the MFCs are coupled with external supercapacitors or batteries to improve the quality of the electric energy. Supercapacitors and batteries store the energy harvested by the MFC buffering discontinuities in the generation and improving power and deliver energy and power to utilities. A recent review showed the possible way to harvest electricity from MFCs^82^. Several examples on energy harvesting and utilization for practical applications are also presented in literature^82–93^.

At the same time, it was shown that intermittent system operation rather than continuous mode are beneficial for harvesting more electricity/power from working MFCs^94,95^. Recently, the supercapacitive properties of the MFCs electrodes were investigated^46,53,96–98^. Malvankar *et al*. in 2012 showed that cytochromes were able to store electrons and therefore the capacitive features of the anodes were shown^99^. Several examples related with the anode supercapacitive properties related to the different utilized materials were freshly presented^100–102^. In parallel, anode and cathode electrodes of a MFC were lately explored as the negative and positive electrodes of an internal supercapacitor (SC-MFC)^46,53,96–98^. The system was therefore studied together not just a single electrode making the SC-MFC suitable for practical applications. In the SC-MFC case, the two electrodes were self-polarized as a result of the red-ox reactions occurring at the electrodes interfaces^46,53,96–98^. The anode was negatively self-polarized thanks to the electro-active bacteria colonization and the creation of anaerobic conditions. The cathode was positively self-polarized due to the air-breathing cathode configuration that provides oxygen and therefore aerobic conditions. Both electrodes were self-polarized without the supply of any external power. Previously studies showed high current/power discharges using a glass bottle membraneless SC-MFC with volume of 0.125 L^53,96–98^. Despite the results were quite promising, the work was a lab scale study due to the small MFCs utilized and the operating conditions adopted^96–98^.

In this current work, the supercapacitive features of the electrode operating as an internal supercapacitor were tested in a scaled up reactor with 1 L empty volume. In fact, a MFCs stack containing 28 MFCs was tested in both standard and supercapacitive mode. The system was a stack of 28 MFCs with all the anodes connected in parallel and with also the cathodes connected in parallel. Electrolytes with seven different solution conductivities were investigated simulating diverse wastewater with different ionic strength. Polarization and power curves are presented and the response of parallel connected electrodes was monitored. Full discharges are also presented and electrochemical parameters such as equivalent series resistance (ESR), maximum power curves, pulses power curves are reported and discussed.

## Results and Discussion

**Figure 1:**
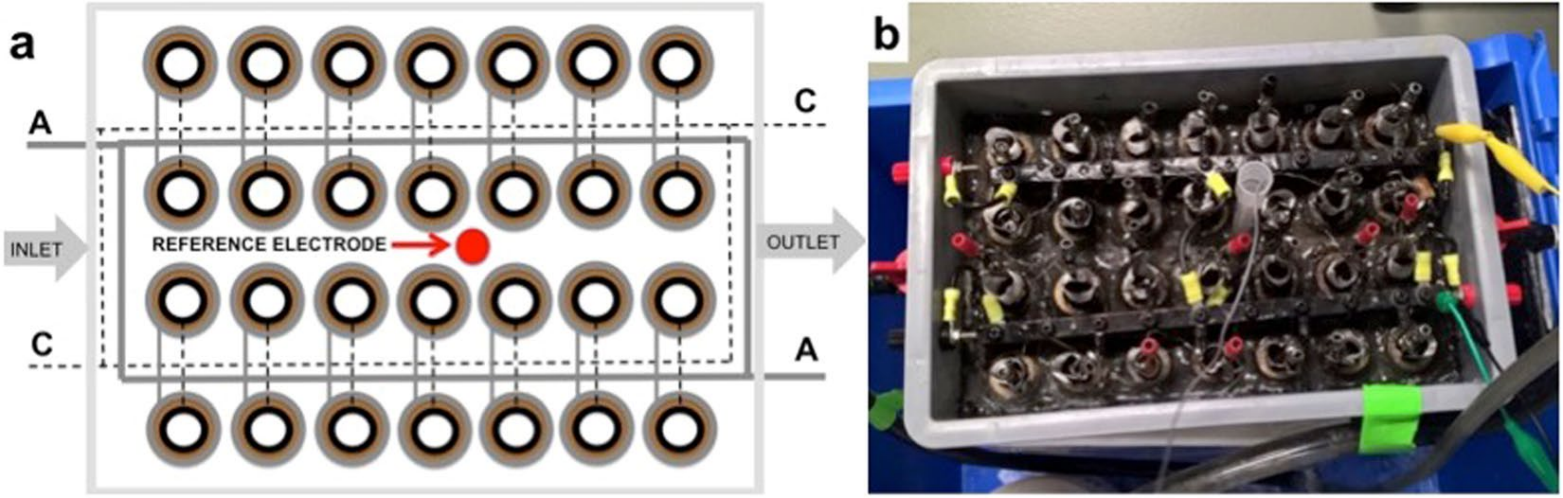
Schematic (**a**) and picture (**b**) of the MFC stack, manufactured in the Bristol BioEnergy Centre, BRL, UWE, UK.

The MFCs stack scheme and the image are shown in Fig. 1. Details on its construction and the materials used are given in the Materials and Method Section. Briefly, the MFCs stack consisted in 28 cylinders MFCs with ceramic separators positioned into a rectangular-shaped plastic box with an empty volume of 1 liter (Fig. 1). Carbon veil anodes were wrapped on the external face of each ceramic cylinder and immersed in the electrolyte. Activated carbon air-breathing cathodes were wrapped on the internal surface of the cylinders and exposed to air. In the schematic of Fig. 1a, straight grey lines indicate the connections of the anodes and dotted black lines indicates the connections of the cathodes. The anodes and cathodes of the MFCs were parallel connected. The reference electrode (Ag/AgCl 3 M KCl) was inserted in the center of the stack (Fig. 1).

### Polarization curves for the MFC operating in standard mode

**Figure 2:**
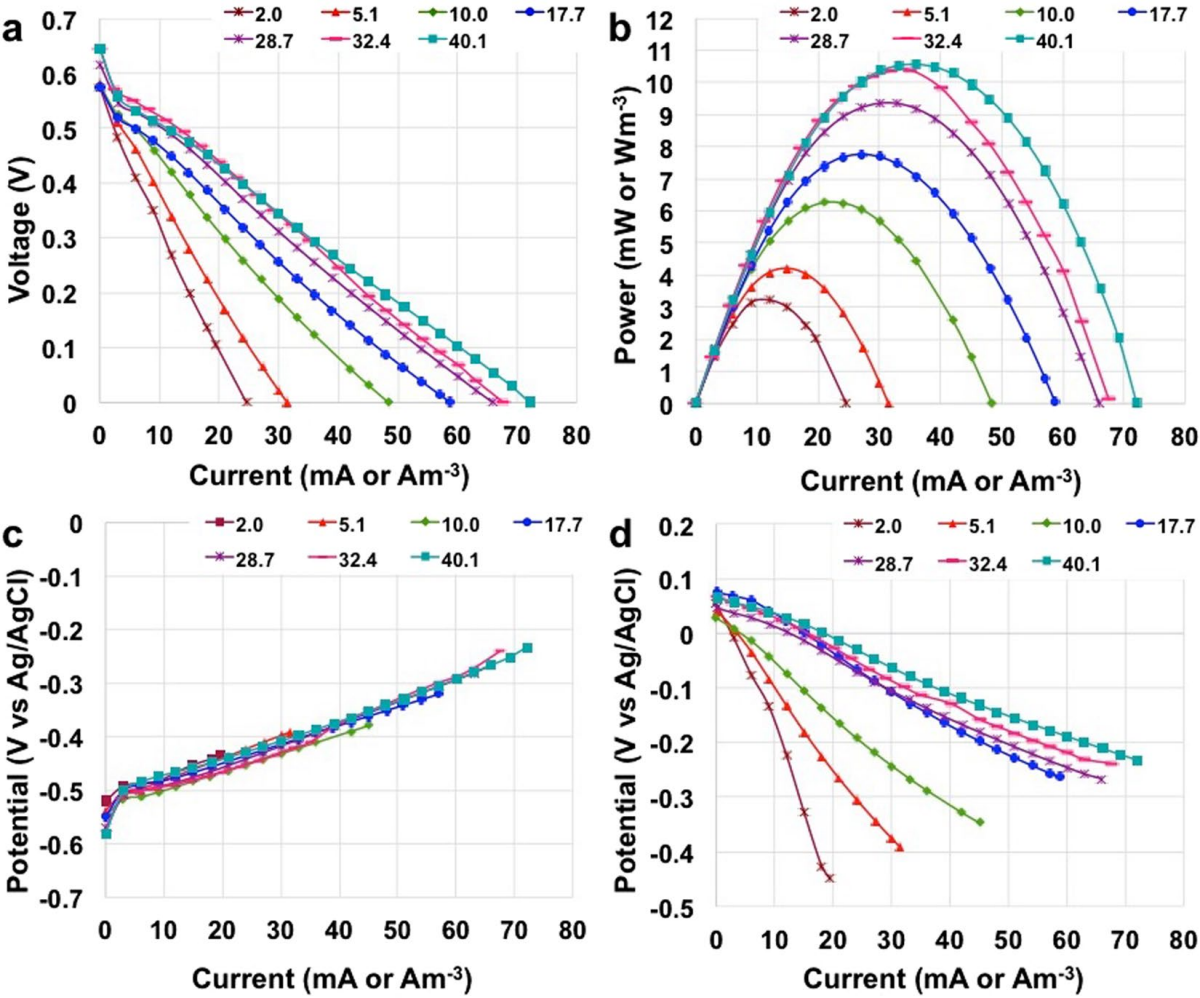
Overall polarization curves (**a**), power curves (**b**), anode polarization curves (**c**) and cathode polarization curves (**d**) of the ceramic MFCs stack at different solution conductivity.

Polarization curves were recorded for each of the solution investigated after that the MFC was stabilized for few days. Interestingly, the electrochemical performance improved with the increase in the electrolyte solution conductivity (Fig. 2a). Similar open circuit voltage (OCV) was measured despite the different electrolytes and quantified in 594 ± 21 mV. On the contrary, the short circuit current (at null voltage) varied significantly with a minimum value of 24.6 mA (24.6 Am^−3^) at a conductivity of 2.0 mScm^−1^ till a maximum value of 72.1 mA (72.1 Am^−3^) at a conductivity of 40.1 mScm^−1^. Remarkably, the shape of the polarization curves can be approximated to a linear V-I trend underlying that ohmic losses still remain the major contribution of MFCs stack losses (Fig. 2a).

The power generation increased with the solution conductivity of the electrolyte (Fig. 2b). The lowest power generation peak was 3.22 mW (3.22 Wm^−3^) at a conductivity of 2.0 mScm^−1^ and the highest was 10.6 mW (10.56 Wm^−3^) at a conductivity of 40.1 mScm^−1^. It must be noted that despite the power generation increased, the enhancements were not directly proportional with the solution conductivity. In fact, when the latter increased 5 fold from 2.0 mScm^−1^ to 10.0 mScm^−1^, the power generation approximately doubled (+94%). When the solution conductivity increased from 10.0 mScm^−1^ to 40.1 mScm^−1^ (4 fold), the power generation increased from 6.26 mW (6.26 Wm^−3^) to 10.56 mW (10.56 Wm^−3^) respectively with a growth rate of ≈70%. Those data allow speculating that doubling the solution conductivity of the electrolyte, the power generated increased by 20%.

During the overall polarization curves, the trend of the anode and cathode electrodes were also monitored and presented in Fig. 2c,d, respectively. It is interesting to notice that both anode and cathode polarization curves are straight lines that are regulated by ohmic losses in which diffusion phenomena do not take place (Fig. 2c,d). The anode trend did not show particular differences within the different electrolyte tested (Fig. 2c). On the contrary, the cathode trend was enhanced significantly with the increase in solution conductivity (Fig. 2d). Electrode potentials depend on the potential of the red-ox processes occurring at the electrode and on the ohmic drop between the electrode and the reference electrode, i.e. the uncompensated resistance (R_U_). The latter in turn depends on the distance between electrode of interest and reference electrode and on the ionic conductivity of the electrolyte. Given that the reference electrode was positioned in the middle of the box, the anode and the cathode potentials are representative of an average value over the different individual cells.

The analysis of the parallel connected anode and cathode potential trends permits to estimate the average value of the anode (R_A_) and cathode (R_C_) resistances that include the different contribution of R_U_ for anode and cathode (see eq. 2 and 3). Considering the anode, R_U_ can be considered low because the anode is immersed in the same electrolyte than the reference electrode. On the contrary, R_U_ for cathodes is expected to be higher because it is affected by the resistance of the ceramic separator. The results showed that the increase in the solution conductivity affects only the cathode presumably because it decreases the ohmic resistance through the ceramic membranes.

### Analysis of full discharge

**Figure 3:**
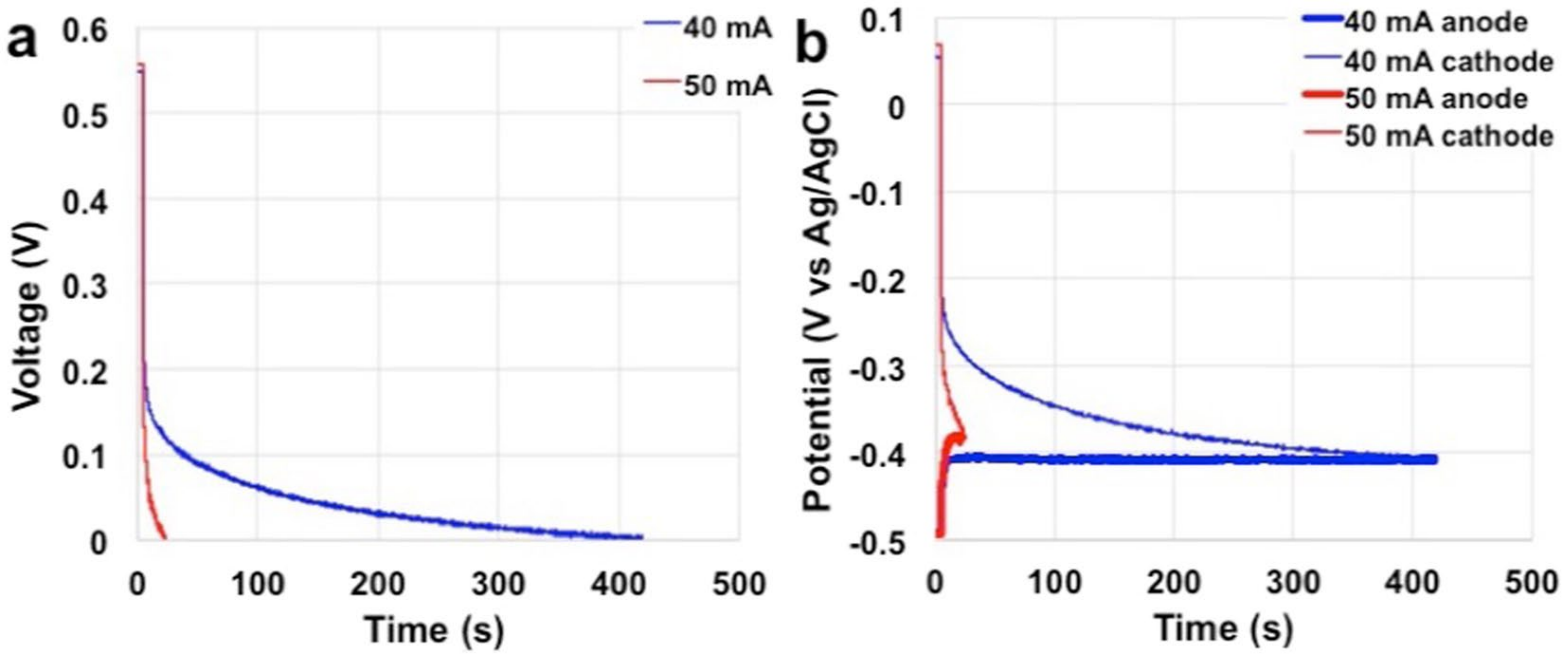
Full discharge at different current levels of the MFCs stack with electrolyte solution conductivity of 5.1 mScm^−1^. Cell voltage (**a**) and parallel connected electrode profiles (**b**).

In order to evaluate how long the box can sustain the maximum current achieved, galvanostatic tests were performed. Full galvanostatic discharge curves are presented in Fig. 3. In fact, overall MFCs stack voltage and parallel connected anode and cathode potential profile are reported versus time at current of 40 mA and 50 mA (40 Am^−3^ and 50 Am^−3^) with electrolyte solution conductivity of 5.1 mScm^−1^ (Fig. 3). At current of 40 mA (40 Am^−3^), the MFCs stack can sustain currents for only roughly 415 s till the MFCs stack voltage reach the null value (Fig. 3). This is mainly due to the very high initial ohmic drop followed by a slow decrease related to the diffusion/mass transport sluggish kinetics of the red-ox processes. At 50 mA (50 Am^−3^), it was not possible to reach stationary potentials and the discharge lasted only 19 s (Fig. 3). The responsible of this trend is the cathode (Fig. 3b). Interestingly, in the case of the highest currents, a voltage transient can be observed at very short time that can be related to the capacitive features of the electrodes (mainly of the cathodes). This allows obtaining power also at high currents before the MFCs stack voltage goes to null value. Therefore, we analyzed these voltage transients applying current of 40 mA (40 Am^−3^) for short times (up to 5 s) and the results are discussed in the next section.

### Discharges for MFCs operating in supercapacitive mode

**Figure 4:**
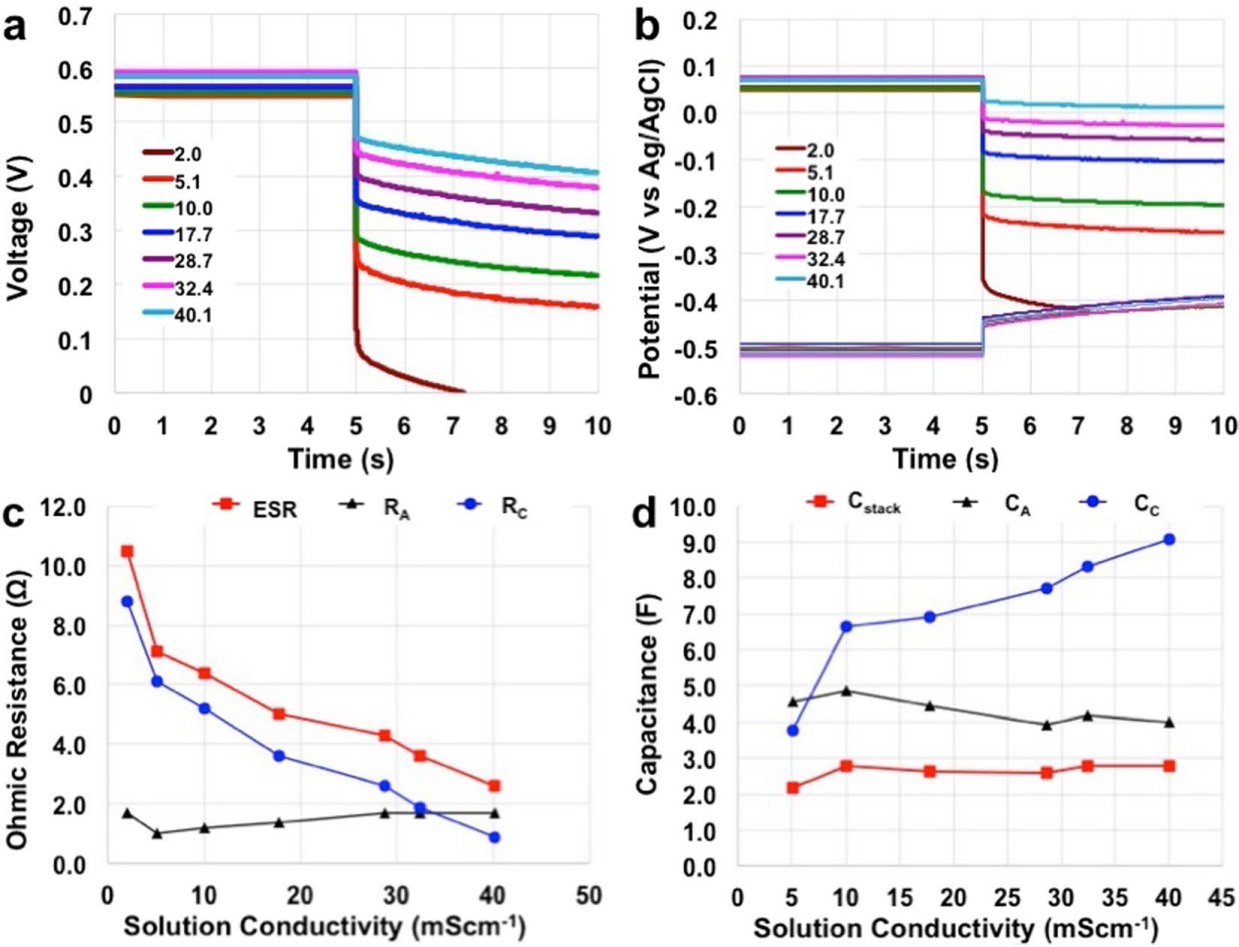
Discharges at i_pulse_ of 40 mA (40 Am^−3^) for t_pulse_ of 5 s after 5 s of rest. Overall cell voltage profile (**a**) and parallel connected anode and cathode potential profiles (**b**). MFCs stack and electrodes ohmic resistance (**c**) and capacitance (**d**).

After the polarization curve was done, the MFC was left for at least 3 hours in OCV till the voltage output was stable. After that, cell discharges were performed with the anode as the negative electrode and the cathode as a positive electrode of the internal supercapacitor. Here are reported and discussed cell discharges for t_pulse_ of 5 s and i_pulse_ of 40 mA (40 Am^−3^) at different electrolyte solution conductivities (Fig. 4a). Discharge profiles of the single electrodes are also presented (Fig. 4b). As it can be noticed, SC-MFC with solution conductivity of 2.0 mScm^−1^ completed the full discharge after 2.1 s (Fig. 4). The OCV of the cell in rest condition ranged between 549 mV and 592 mV (average of 570 ± 17 mV) (Fig. 4a). Cathode open circuit potential (OCP) in rest ranged between + 47 mV (vs Ag/AgCl) and + 76 mV (vs Ag/AgCl) (average of 62 ± 12 mV) (Fig. 4b). Anode OCP in rest ranged between −493 mV (vs Ag/AgCl) and −520 (vs Ag/AgCl) (average of −508 ± 9 mV) (Fig. 4b).

It can be noticed a decrease in the ESR with the increase in the solution conductivity as underlined in Fig. 4a and better described in Fig. 4c. Particularly ESR had its highest value of 10.5 Ω at solution conductivity of 2.0 mScm^−1^ and its lowest value of 2.6 Ω at solution conductivity of 40.1 mScm^−1^ (Fig. 4c). As the anode profiles were similar in the different conditions investigated (Fig. 4b), the major difference was detected within the cathode profiles (Fig. 4b). In fact, a decrease in the ohmic drop in the cathode profile during the discharge was detected (Fig. 4b). R_C_ decreased constantly from 8.8 Ω (at solution conductivity of 2.0 mScm^−1^) to 0.9 Ω (at solution conductivity of 40.1 mScm^−1^) (Fig. 4c). Those data are in agreement with the discussion related to R_U_ through the ceramic separator that is expected to decrease with the increase in solution conductivity. Interestingly, R_A_ was approximately stable within the electrolyte investigated ranging between 1.0 Ω and 1.7 Ω (Fig. 4c). The overall capacitance of the MFC and the capacitances of the single electrodes were also calculated (Fig. 4d, eqs 5 and 6). Considering the full discharge at t_pulse_ of 5 s, C_stack_ had its lowest value of 2.2 F at solution conductivity of 5.1 mScm^−1^. C_stack_ increased with the electrolyte ionic strength till solution conductivity of 10.0 mScm^−1^ then a plateau was reached with C_stack_ of roughly 2.8 F (Fig. 4d).

### Power curves for supercapacitive MFCs

**Figure 5:**
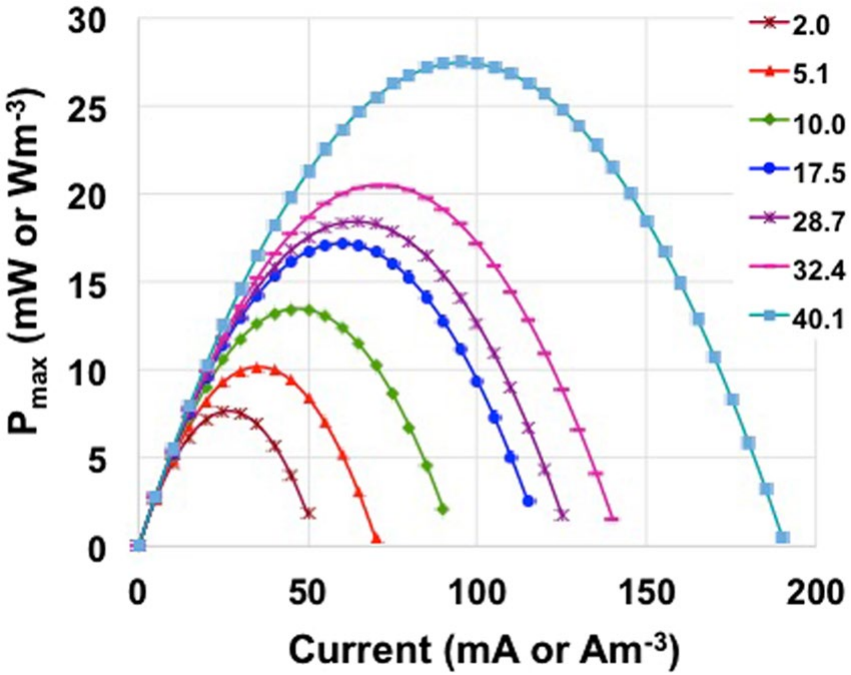
P_max_ curves at different electrolyte solution conductivity and currents.

**Figure 6:**
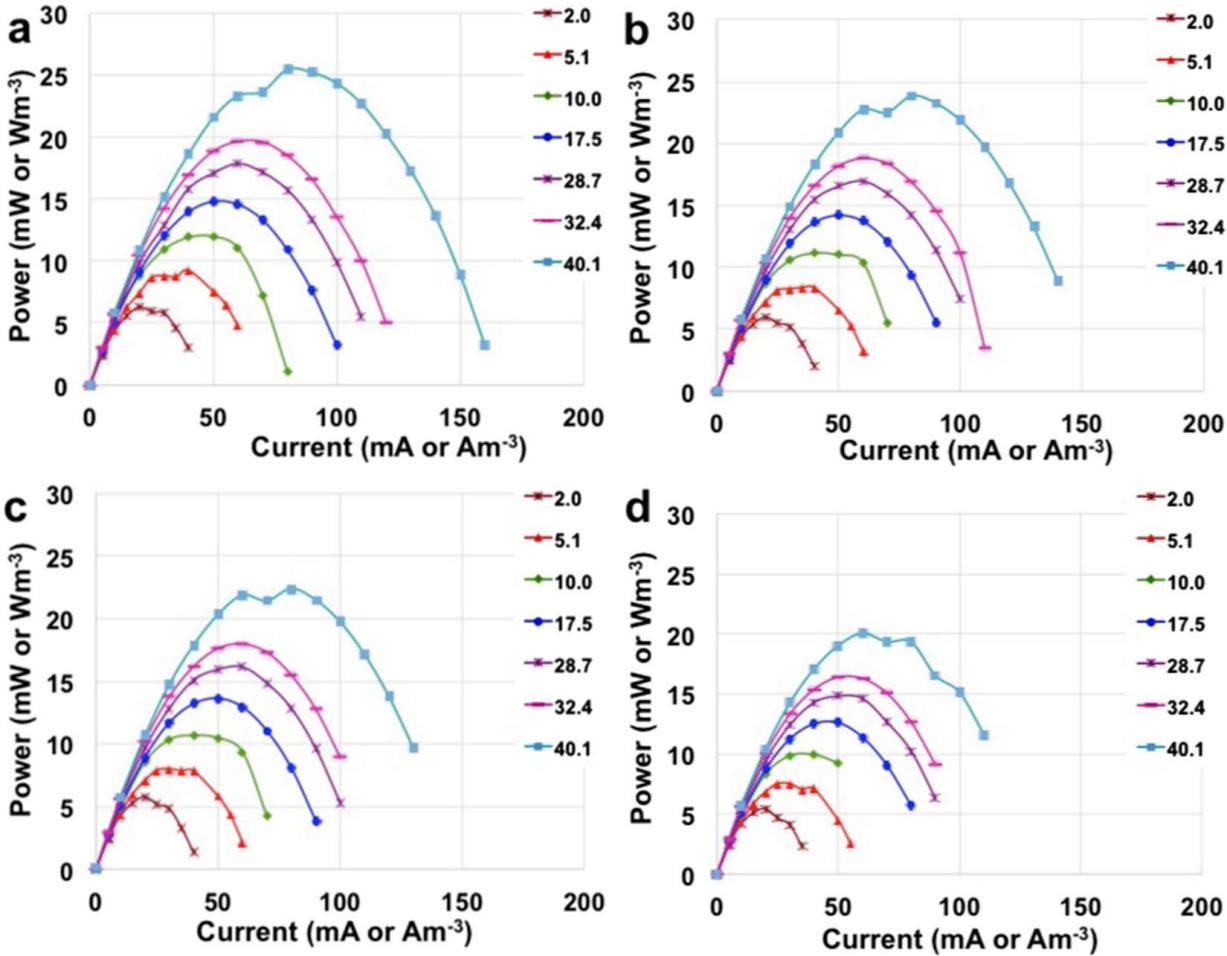
P_pulse_ curves at t_pulse_ of 0.2 s (**a**), 1 s (**b**), 2 s (**c**) and 5 s (**d**) for the SC-MFC at different electrolyte solution conductivity and currents.

Power curves are here presented in terms of maximum power achievable (P_max,_ eq. 7) (Fig. 5) and of pulse power (P_pulse_, eq. 8) obtained with t_pulse_ of 5 s, 2 s, 1 s and 0.2 s respectively (Fig. 6). P_max_ increased with the electrolyte solution conductivity having a power peak measured of 7.6 mW (7.6 Wm^−3^), 10.2 mW (10.2 Wm^−3^), 13.5 mW (13.5 Wm^−3^), 17.2 mW (17.2 Wm^−3^), 18.4 mW (18.4 Wm^−3^), 20.5 mW (20.5 Wm^−3^) and 27.4 mW (27.4 Wm^−3^) for solution conductivity of 2.0 mScm^−1^, 5.1 mScm^−1^, 10.0 mScm^−1^, 17.5 mScm^−1^, 28.7 mScm^−1^, 32.4 mScm^−1^ and 40.1 mScm^−1^ respectively (Fig. 5). P_pulse_ were respectively lowering compared to P_max_ due to the presence of the capacitive features of the electrode  that determines the decrease of voltage over time. Also in this case, the power produced increased with the ionic strength. At the lowest solution conductivity (2.0 mScm^−1^), the peak of power recorded was 6.3 mW (6.3 Wm^−3^), 6.0 mW (6.0 Wm^−3^), 5.8 mW (5.8 Wm^−3^) and 5.6 mW (5.6 Wm^−3^) for t_pulse_ of 0.2 s, 1 s, 2 s and 5 s respectively. At highest solution conductivity (40.1 mScm^−1^) instead, the peak of power recorded was 25.4 mW (25.4 Wm^−3^), 23.8 mW (23.8 Wm^−3^), 22.4 mW (22.4 Wm^−3^) and 20.0 mW (20.0 Wm^−3^) for t_pulse_ of 0.2 s, 1 s, 2 s and 5 s respectively. A 20-fold increase in solution conductivity from 2.0 mScm^−1^ to 40.1 mScm^−1^ enhanced the performances four times.

### Comparison in performances between MFC and SC-MFC

**Figure 7:**
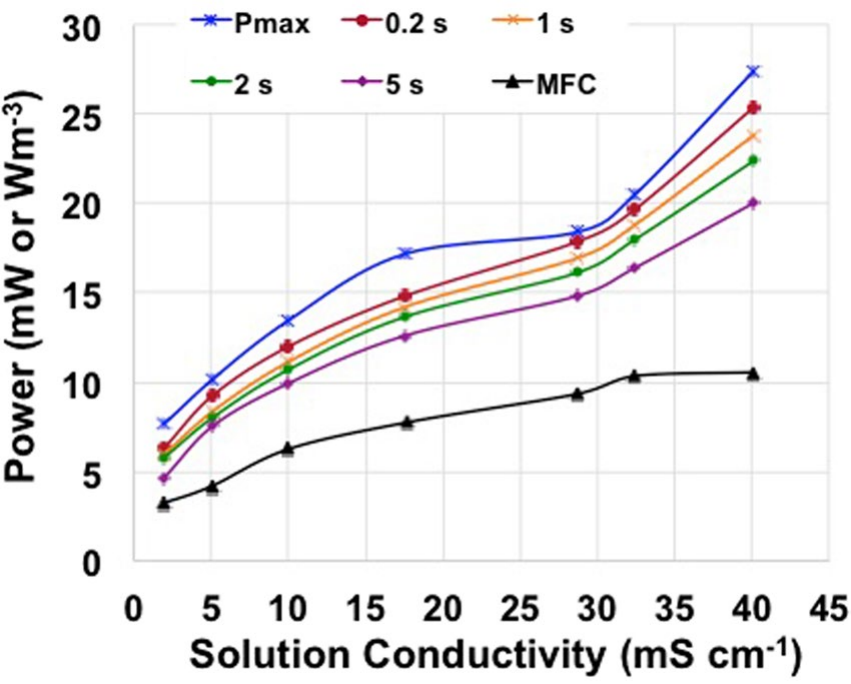
P_max_, P_pulse_ at t_pulse_ of 0.2 s, 1 s, 2 s and 5 s in supercapacitive mode and MFC power peaks in standard mode at different electrolyte solution conductivity.

Figure 7 shows the trend of increasing power peaks with the solution conductivities. Power obtained by the MFC working in supercapacitive (pulse) mode was more than double than that achieved by stationary traditional MFC working mode, and therefore more attractive for real applications.

### Outlook and perspective

The performances of the MFCs stack tested in standard and supercapacitive mode are here presented. This work is one of the few studies presented in literature performed with a system with a volume greater than few mL but actually on a liter scale^103–107^. By far, this is the first time that a MFCs stack with empty volume of 1 L in supercapacitive mode is tested. In fact, this is a preliminary attempt on scaling up MFCs at larger scale that laboratory scale (volume lower than 50 mL). This exact ceramic MFCs stack was previously investigated with human urine in real experimental conditions, real field trials applications and previously presented^108^. Differently than before, in this case, the solution conductivity of the working electrolyte was changed in a significant range from 2.0 mScm^−1^ to 40.1 mScm^−1^ simulating various wastewaters for different applications. Electrochemical performances of the systems in low solution conductivity (such as activated sludge or raw wastewater) till medium-high solution conductivity (such as human urine and industrial wastewater) were investigated. As it usually occurs in MFCs systems, the performances were limited by the electrolyte conductivity. The increase in solution conductivity led to a significant decrease in the ohmic losses of the system that was beneficial for the power output of the SC-MFC. To enhance the performances output, this system should be utilized in high solution conductivity organic waste such human urine that has a very high natural ionic strength often above 50 mScm^−1^ after hydrolysis^108,109^.

In this experimentation, the power produced in terms of volumetric power was lower compared to previously presented literature^71–77^. This was due to the reactor size that was much larger than commonly used lab-scale reactor with volume lower than 50 mL^71–77^. Recent reviews on power generation achieved in MFCs showed that as the reactor becomes larger, lower the volumetric power is recorded^71–73^. In fact, literature showed that MFCs system can achieve above 10000 Wm^−3^ in μL volume scale^77^ but with the increase in volume over 1 L scale the power dropped to below 100 Wm^−3^ ^71–77^. This might be due to less compact design with increase of space between anode and cathode (and consequent increase in ohmic losses), losses due to series/parallel connections and increase in empty volumes in which parasitic reactions (e.g. fermentation) can occur. In the MFC field, the reactor scalability remains an issue to be addressed for large operations and further large-size design must be introduced and studied. Volumetric power is a very important parameter to consider when compared the electrochemical performances of one system with the existing literature but from the practical application point of view, the overall power and not the power referred to volume or electrode surface area must be considered. Unfortunately, this point of view is often forgotten and/or regularly criticized. In fact, the absolute power output as well as operating current and voltage are the parameters that have to be carefully considered for powering practical applications. In this case, MFCs were tested in traditional way utilizing anode and cathode as electrodes and in supercapacitive way utilizing the anode and cathode of the MFC as the negative and positive electrode of an internal supercapacitor. The overall and volumetric power produced by the supercapacitive MFC was more than double compared to the one produced by a traditional MFC. This indicated that pulse mode is more appealing for short but more powerful utilization. It was shown before that intermittent working operation is actually more valuable and advantageous in terms of energy produced that could actually be successfully harvested^94,95^.

Future work should focus on the investigation of long terms discharge and self-recharge operations and explore the possibility of utilizing this type of system as simpler energy harvesting devices.

### Summary

Ceramic-based MFCs stack with a total volume of one liter was investigated as traditional MFC and in supercapacitive mode. Seven different electrolyte solution conductivities in the range between 2.0 mScm^−1^ to 40.1 mScm^−1^ were investigated in order to simulate various wastewaters with diverse ionic strenght. Generally, the power output increased with the increasing of the electrolyte solution conductivity due to the reduction in ohmic losses of the overall system. MFC maximum power was achieved with an electrolyte solution conductivity of 40.1 mScm^−1^ measuring 10.6 mW (10.6 Wm^−3^). The system operating in supercapacitive mode boosted up even more the power obtained that registered a maximum of 27.4 mW (27.4 Wm^−3^) at 40.1 mScm^−1^. Operating the system in supercapacitive mode allow to gain roughly 3 times the power obtained and therefore it looks like an interesting pathway to follow to enhance power produced for better energy harvesting and practical applications.

## Materials and Method

### Microbial Fuel Cells Stack Construction and Electrodes materials

Ceramic separator MFCs stack with an empty volume of 1 liter was used for this experimentation (Fig. 1). This kind of MFC was previously described^108,109^. The MFCs stack was designed and built at the Bristol BioEnergy Centre (Bristol, UK) and tested at the University of New Mexico (Albuquerque, NM). Briefly, 28 ceramic cylinders (open on one side) were positioned into a rectangular-shaped plastic box. The ceramic cylinders have a height of ≈4 cm, an internal diameter of ≈2 cm and an external diameter of ≈2.3 cm. Therefore, the ceramic separator was thick ≈0.3 cm. Carbon veil (30 g m^−2^) with geometric area of 240 cm^−2^ was folded and wrapped on the external face of each ceramic cylinder. The carbon veil worked as anode electrode. On the internal face, an air-breathing cathode based on activated carbon as catalyst was utilized. Particularly, activated carbon (AC, Norit SX Ultra, Sigma Aldrich), carbon black (CB, Alfa Aesar) and polytetrafluorethylene (PTFE, 60 wt% solution, Sigma Aldrich) were mixed together into a blender and grinded for few minutes. AC was the catalyst, CB was used to enhance the electrical conductivity of the cathodes as showed previously^110^, PTFE was used as binder and as hydrophobic agent to create a three phase interface (TPI) within the electrode. AC, CB and PTFE mixture in percentage weight of 70%, 10% and 20% was then accommodated on a stainless steel mesh used as current collector and then pressed manually using a roller pin. The cathodes were then cut and put in contact with the internal phase of the cylinder. A hard plastic cover was used to fix the cylinders making sure that the cylinders were not in contact and guaranteeing enough space to avoid clogging during operations. Specific holes were fabricated to leave the internal part of the cylinders open to air allowing the cathodes to be exposed to the atmosphere. On the contrary, the hard plastic cover was hermetically (air-tight) sealed with the rectangular plastic box. The anodes were in fact voluntarily not exposed to the atmosphere. Each cylinder had an anode and a cathode. All the anodes electrodes were connected in parallel and the same was done to the cathodes. Series connection was not possible due to the fact that the electrodes were sharing the same electrolyte. The reference electrode (Ag/AgCl 3 M KCl) was inserted into the box as shown in the scheme and in the image of Fig. 1.

### Microbial Fuel Cells operation

The ceramic MFC stack was inoculated using activated sludge collected from Albuquerque SouthEast Water Reclamation Facility, Albuquerque, NM, USA. Experiments were started when a constant voltage was achieved. Initially, resistance was kept at 1000 Ω and then reduced to 100 Ω. The external resistance was further reduced to 33 Ω and left to this value during the experiments. The solution conductivity was varied from 2.0 mS cm^−1^ to 40.1 mS cm^−1^. Solution conductivity of 2.0 mS cm^−1^ was measured in the case of utilization of activated sludge. Solution conductivity was increased step by step adding potassium phosphate buffer solution (K-PB) and potassium chloride (KCl) and sodium acetate (NaOAc). The solution conductivities investigated are 2.0, 5.1, 10.0, 17.7, 28.7, 32.4, 40.1 mS cm^−1^. Once the solution conductivity was changed, the MFC was put under 33 Ω external resistance for at least two days and then left overnight disconnected before running electrochemical experiments. Four liters electrolyte reservoir was used and the electrolyte was recirculated inside the MFC with a flow rate of 20-22 mL min^−1^. Peristaltic pump (MasterFlex 7523, ColePalmer) was used to keep the continuous flow within the reactor.

### Standard mode operation

MFC polarization curves were taken after leaving the MFC in open circuit voltage (no resistance connected) overnight. Two separate potentiostats (SP-50 Biologic) were used during the polarization curve. The first one was used in two-electrodes mode with working channel connected to the cathode and counter electrode (short-circuited with the reference channel) connected to the anode. The second one was used to measure the potential of the anodes and cathodes both connected in parallel vs the reference electrode potential during the polarization tests. Linear sweep voltammetry was run between OCV and 0 mV at a scan rate of 0.2 mVs^−1^.

### Supercapacitive mode operation

After the polarization curve, the MFCs stack was left in open circuit potential for over 4 hours till the OCV was stable before applying discharges. Galvanostatic discharges were done using a potentiostat (SP-50 Biologic) connecting the cathode to the working channel, the anode to the counter electrode channel and a Ag/AgCl 3 M KCl to the reference channel. The MFC was left in open circuit voltage (OCV) condition then discharges for a certain amount of time (**t**_**pulse**_) and at a certain applied current (**i**_**pulse**_) were done.

### Galvanostatic discharge profiles analysis

In order to characterize in depth the supercapacitive properties of the system, several parameters have to be considered. Firstly, the initial voltage in which the MFC stack is left before applying a discharge is named V_max,OC_. When the discharge occurs, a vertical fall is observed (ΔV_ohmic,stack_) and this is caused by the ohmic resistance of electrodes and electrolyte. The ohmic resistances can be quantified through the equivalent series resistance (ESR) that is calculated through the following equation (eq. 1):

1 $$ESR=\frac{{\rm{\Delta }}{V}_{ohmic,cell}}{{i}_{pulse}}$$

During discharge, parallel connected anode and cathode potential profiles can be measured individually. The analysis of the parallel connected anode and cathode potentials permit to estimate the average value of the anode (R_A_) and cathode (R_C_) resistances that include the different contribution of uncompensated resistance (R_U_) for anode and cathode. R_A_ and R_C_ can be estimated according with equation 2 (eq. 2) and equation 3 (eq. 3):

2 $${R}_{A}=\frac{{\rm{\Delta }}{V}_{ohmic,anode}}{{i}_{pulse}}$$

3 $${R}_{C}=\frac{{\rm{\Delta }}{V}_{ohmic,cathode}}{{i}_{pulse}}$$

As mentioned before, the SC-MFC started at V_max,OC_ and lost immediately ΔV_ohmic,stack_ reaching a voltage value named V_max_ (eq. 4).

4 $${V}_{max}={V}_{max,OC}-{\rm{\Delta }}{V}_{ohmic,stack}$$

After the ohmic losses, the voltage decreases over time due to the capacitive features of the electrodes and the overall system. This voltage variation is termed capacitive variation (ΔV_capacitive,stack_). The cell capacitance (C_stack_) can be calculated according to eq. 5:

5 $${C}_{stack}=\frac{{i}_{pulse}}{s}=\frac{{i}_{pulse}}{\frac{dV}{dt}}$$

in which *s* is the slope of the voltage change over time (dV/dt).

Once again, when electrodes are considered separately, the anode capacitance (C_A_) and the cathode capacitance (C_C_) can be measured independently. In fact, the electrode capacitance is the ratio between the i_pulse_ and the slope of the respective potential profile over the time considered (t_pulse_).

C_A_ and C_C_ can also be correlated to the C_stack_ according with eq. 6:

6 $${C}_{stack}={(\frac{1}{{C}_{A}}+\frac{1}{{C}_{C}})}^{-1}$$

Other electrochemical parameters of interest that describes SC-MFCs are certainly power and energy. The maximum power abbreviated as P_max_, is calculated according with eq. 7:

7 $${P}_{max}={V}_{max}\,\times {i}_{pulse}\,$$

P_max_ corresponds to the highest value that can be obtained considering ohmic losses but neglecting capacitive behaviour of the SC-MFC. The power of a pulse (P_pulse_) obtained during a pulse of a t_pulse_ is lower than P_max_ due to the consideration of the capacitive behaviour that occurs during the discharge. P_pulse_ can be calculated according with eq. 8:

8 $${P}_{pulse}=\frac{{i}_{pulse}\,{\int }_{0}^{t}Vdt}{{t}_{pulse}}=\frac{{E}_{pulse}}{{t}_{pulse}}$$

P_pulse_ is the ratio between the energy that is delivered during the pulse (E_pulse_) and the time (t_pulse_) for which the pulse occurs. Power is reported in mW and Wm^−3^ considering an operating volume of 1 L.
